# Surgery

**Published:** 1894-04

**Authors:** 


### *SELECTIONS AND ABSTRACTS.*

---

### SURGERY.

EDITED BY DR. F. W. McRAE.

### TREATMENT OF ERYSIPELAS WITH HYPODERMATIC INJECTIONS OF CARBOLIC ACID.

Dr. J. McFadden Gaston, of Atlanta, contributed to the *Medical and Surgical Reporter* an article on this subject:

About twenty years ago my attention was directed to this remedy by a report of successful results in an English medical journal, and, finding that it fulfilled all that was claimed for it, I have employed it frequently in all the various forms of erysipelas which have come under my care. It has been attended with invariably good effects in promptly arresting the progress of this disease. In some cases no other treatment has been employed, so as to test its virtues without resorting to constitutional measures, and the effect has been entirely satisfactory.

I recall only a single case during all this period, which did not yield to this treatment, and that was in a patient, to whom I was called in consultation, at an advanced stage of erysipelas involving the entire scalp, accompanied with coma. As an offset to this, I have frequently employed the carbolic acid hypodermatically when the scalp was partially involved in erysipelas, with complete relief.

I am reminded by an allusion of Dr. W. H. Wathen to complications of delivery at term with erysipelas, of a case in which there was a face presentation, requiring podalic version, and followed by erysipelas of the genitalia and the hypogastric region. The injections, hypodermatically, of carbolic acid in various portions of the area affected was crowned with complete success.

The formula employed by the original reporter, whose name is not remembered, was a solution of carbolic acid in dilute alcohol.

But this was modified by me afterwards, and I have used during the past ten years the following:

> ℞ Carbolic acid.......................................f℥ i.

> Glycerine ..............................................f℥ iii.

> Distilled water.........................................f℥ iv.

>

> Mix and inject hypodermatically one syringeful in each portion of the size of a hand, daily.

It will be observed that this is a twelve and a half per cent. solution, and only in a few cases has any local irritation resulted from the injection.

When the thickened and hardened condition of the skin has rendered it difficult to introduce the needle, I have selected points on the border of the dermatitis to make the injection so as to reach the areolar tissue beneath the skin. There is little pain connected with it. I am convinced that many cases of erysipelas will yield to this treatment; but it does not preclude the resort to any internal means which may be indicated, and when the extent of surface involved is large, or the febrile condition is great, I have used internal remedies.

The best effects have been secured by giving at the outset calomel, followed by Epsom salts in senna tea, to procure free evacuations from the bowels. After purgation I have given twenty-five drops of the muriated tincture of iron every three hours until an ounce is taken. In some cases chlorate of potash in ten-grain doses has been combined with the iron, especially when there is a tendency to the phlegmonous form of erysipelas.

But when I am called early to the case, and find the erysipelas circumscribed, I rely upon the hypodermatic use of the formula given above and rarely have any occasion to employ other means of cure.

An instructive case was presented a few years after commencing the use of this remedy. Having treated an acute case of erysipelas in the family of a man, he informed me that he was subject to a periodic recurrence of this disease in his feet and legs every spring. I had an understanding with him to give me notice of the next appearance, with a view to test the remedy in this chronic state. In the course of time I was called, and found a well developed case of erysipelas on both legs, extending up to the knees. The carbolic acid was used hypodermatically in two places on each leg, and repeated on two successive days, without any other treatment. All traces of the disease disappeared, and during five years subsequently there was no return of the disease, nor did there seem to be any threatening of future trouble.

In this instance the elimination of disease was complete and indicates a specific action of carbolic acid in the cure of erysipelas.

The influence upon the system of such an injection is general and not confined to the immediate location of the puncture, so that it may be considered as a constitutional impression of the remedy. Some caution is requisite against an undue quantity of this solution of carbolic acid being used on one occasion, lest a toxic influence should be manifested. But I have repeatedly introduced a syringeful of the solution in four different places, without producing any untoward effect, and hence it may be allowed that this quantity, distributed over an area of four hands' breadth, is entirely safe. It is also found proper to repeat the injections daily for three days, but I have never had any occasion in which it was requisite to continue the treatment for a longer time. My patients have generally been so far relieved within three days, or less time, as to dispense with further injections.

Since the above was written Dr. Gaston has treated a case of erysipelas of the right ear and neck, involving the cheek and eye, by the injection of carbolic solution in two points. The prompt arrest of the disease in twenty-four hours was secured.

---

### The Treatment of Malignant Tumors by Inoculations of Erysipelas.

Dr. William B. Coley (*American Journal of the Medical Sciences*) while collecting the cases of sarcoma treated at the New York Hospital during the past fifteen years, found a case that seemed convincing evidence that erysipelas possessed a powerful curative principle antagonistic to sarcoma.

Five operations had been performed within a space of three years. At the last operation it was found impossible to remove all of the tumor, and the case was considered hopeless. Two weeks after the operation a severe attack of erysipelas occurred, followed by a second attack shortly after the first had subsided. During the progress of the erysipelas the remains of the sarcoma entirely disappeared, the wound rapidly healed, and the patient remained well seven years afterward. The diagnosis in this case had been repeatedly confirmed by well known pathologists, and there was no possibility of attributing the cure to any other cause than the erysipelas.

If erysipelas, a disease produced by a specific organism, could cure a case of undoubted sarcoma when occurring accidentally, it seemed fair to presume that the same benign action would be exerted in a similar case if erysipelas could be artificially produced.

Dr. Coley applied treatment to ten cases which he has reported in detail. In addition to his own cases he has collected and tabulated all the reported cases of carcinoma and sarcoma in which erysipelas, either spontaneous or artificial, intervened. It is upon a careful study and analysis of these cases, as well as upon the more practical experience derived frcm his own cases, that his conclusions are based.

We find a total of thirty-eight cases of malignant disease in which an erysipelas has occurred, either by accident or intent. Of these thirty-eight cases the erysipelas occurred accidentally in twenty-three cases, and was the result of inoculation in fifteen cases (including Dr. Coley's own); seventeen cases were carcinoma, seventeen cases were sarcoma, four either sarcoma or carcinoma. The immediate and final results were as follows:

*Carcinoma.*—Of the seventeen cases three were permanently cured. In addition, one case of probable carcinoma was well five years after the attack of erysipelas. Of the remaining thirteen, ten showed improvment, which, although temporary, undoubtedly added to the life of the patient in most cases. One case died, as a result of the erysipelas, on the fourth day.

*Sarcoma.*—In sarcoma the curative action of the erysipelas was even more marked. Of the seventeen cases of sarcoma we find seven, or forty-one per cent., well and free from recurrence from one to seven years after the attack of erysipelas. In addition to these seven cases there is a probable sarcoma of the breast, that was cured.

Ten of the remaining eleven showed more or less marked improvement, in some cases the tumor entirely disappearing, and not recurring for several months. One case died as a probable result of the erysipelas which was in this instance accidental.

In nearly every instance the tumor was not a primary growth, amenable to operative treatment, but either a recurrence after operation had been tried and failed, or from its nature inoperable.

*Conclusions.*—1. The curative effect of erysipelas upon maligdant tumors is an established fact.

2. The action upon sarcoma is more powerful than upon carcinoma, in about the ratio of three to one.

3. The treatment of inoperable malignant tumors by repeated inoculations of erysipelas is both practicable and not attended with great risk.

4. The curative action is systemic, and is probably due chiefly to the toxic products of the streptococcus, which products may be isolated and used without producing erysipelas.

5. This method should not be employed indiscriminately until further experiments have proved its limitations.—*The Philadelphia Polyclinic.*

---

### BORIC ACID FOR BOILS.

Alison claims good results in general furunculosis by giving from ten to fifteen grains of boric acid for eight or ten days, divided into two doses daily. At the same time, four or five times a day, the inflamed areas are washed with a hot solution of boric acid in the strength of four per cent. Between the applications of this lotion, compresses are applied to the diseased parts, which had been wet with the same solution of boric acid. By this means the author thinks it possible to avoid surgical interference.—*The American Therapist.*

